# Application of Fe-MOFs in Photodegradation and Removal of Air and Water Pollutants: A Review

**DOI:** 10.3390/molecules28207121

**Published:** 2023-10-17

**Authors:** Jun Cai, Yang Peng, Yanxin Jiang, Li Li, Hua Wang, Kongzhai Li

**Affiliations:** 1 National Joint Engineering Research Center of Energy Saving and Environmental Protection Technology in Metallurgy and Chemical Engineering Industry, Kunming University of Science and Technology, Kunming 650093, China; caijun0117@kust.edu.cn; 2 Kunming Electric Power Design Institute Limited Liability Company, Kunming 650034, China; 3 Yunnan Hubai Environmental Protection Technology Co., Ltd., Kunming 650034, China; 4 Zhejiang Ecological and Environmental Monitoring Center, Hangzhou 310012, China; 5 State Key Laboratory of Complex Nonferrous Metal Resources Clean Utilization, Kunming University of Science and Technology, Kunming 650093, China

**Keywords:** Fe-MOFs, photocatalysis, water substances, gas substances

## Abstract

Photocatalytic technology has received increasing attention in recent years. A pivotal facet of photocatalytic technology lies in the development of photocatalysts. Porous metal–organic framework (MOF) materials, distinguished by their unique properties and structural characteristics, have emerged as a focal point of research in the field, finding widespread application in the photo-treatment and conversion of various substances. Fe-based MOFs have attained particular prominence. This review explores recent advances in the photocatalytic degradation of aqueous and gaseous substances. Furthermore, it delves into the interaction between the active sites of Fe-MOFs and pollutants, offering deeper insights into their mechanism of action. Fe-MOFs, as photocatalysts, predominantly facilitate pollutant removal through redox processes, interaction with acid sites, the formation of complexes with composite metal elements, binding to unsaturated metal ligands (CUSs), and hydrogen bonding to modulate their respiratory behavior. This review also highlights the focal points of future research, elucidating the challenges and opportunities that lie ahead in harnessing the characteristics and advantages of Fe-MOF composite catalysts. In essence, this review provides a comprehensive summary of research progress on Fe-MOF-based catalysts, aiming to serve as a guiding reference for other catalytic processes.

## 1. Introduction

In the wake of the continuous development of the global economy and the rapid expansion of industry and agriculture activities, a variety of environmental and energy challenges has grown increasingly severe. These environmental pollutants, encompassing waterborne and atmospheric contaminants, pose significant threats to public health [1,2]. These pollutants can be broadly categorized into two main types: natural pollutants and anthropogenic pollutants, with the latter being the primary culprits behind public health hazards. Water pollutants encompass a diverse array, including antibiotics, dye wastewater, heavy metals, pesticides, polycyclic aromatic hydrocarbons, pharmaceuticals, and PPCPS, while air pollutants include carbon oxides, nitrogen oxides, hydrocarbons, and VOCs [3,4,5,6,7,8,9,10,11,12,13]. Conventional treatment methods, such as physical adsorption, chemical oxidation, and biological treatment, are unable to meet the requirements of efficient, economical, and environmentally friendly treatment of such pollutants. Therefore, the green, cost-effective, and efficient photocatalytic approach has emerged as a standout solution. Photocatalytic oxidation, relying on semiconductor materials, leverages the abundant solar energy available in nature as its driving force to decompose pollutants. The chief advantage of this technology lies in its ability to achieve complete pollutant degradation within environmental media at ambient temperature and pressure. As a result, pollutants are rendered relatively environmentally friendly and more amenable to subsequent treatment [14,15,16,17,18,19,20,21].

Metal–organic frameworks (MOFs) are polycrystalline coordination polymers featuring a multi-dimensional stereo-structure, formed by amalgamation of metal ions (or clusters of metal oxides) with organic ligand molecules containing elements such as carbon, nitrogen, and oxygen through coordination bonds [22,23,24,25,26,27,28]. In comparison to other commonly used photocatalysts, MOF materials boast a series of distinctive advantages: (1) Both metal nodes and organic linkers within MOFs play pivotal roles in photocatalytic reactions. It has been reported that metal nodes and organic linkers can be regarded as isolated semiconductor quantum dots and light-absorbing antennae, respectively. Under light irradiation, metal nodes can be directly excited or activated by organic linkers; (2) MOFs exhibit high porosity and a significant specific surface area, facilitating the exposure of a greater number of reaction sites and promoting the transport of substrates and products; (3) the structure of MOFs is adjustable, allowing for the expansion of the optical response range, and the theoretical bandgap of MOFs falls within the range of 1–5.5 eV; (4) the porous structure of MOFs creates shorter pathways for charge carriers to reach the system and engage with substrates. This aids in enhancing the separation of photogenerated electrons from holes; (5) metal cations exhibit diverse coordination chemistry, and there exists a multitude of organic connectors. Reasonable selection of metal ions and organic ligands can modify their light absorption properties, enabling the efficient utilization of sunlight, among other advantages [29,30,31,32].

**Figure 1:**
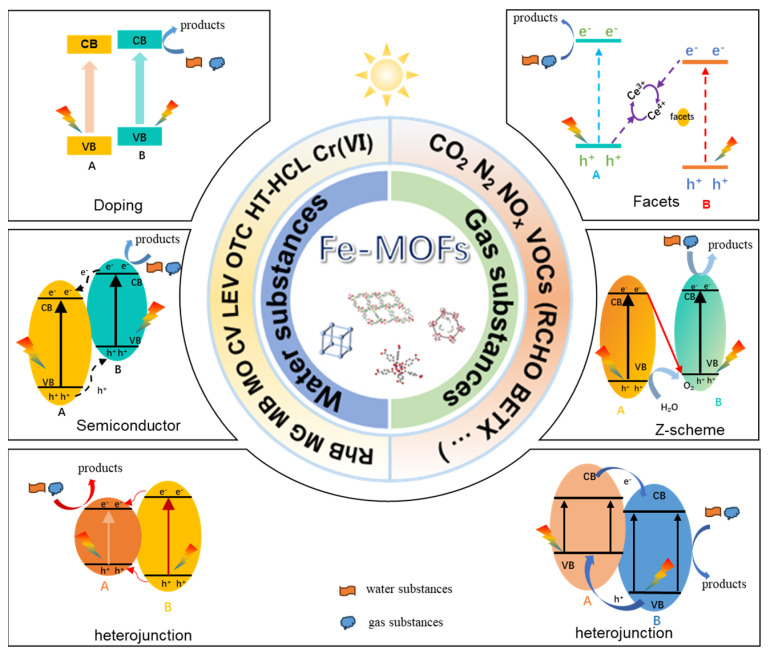
Fe-MOFs for water and gas pollutant photodegradation (A and B represent two different Fe-MOFs materials or Fe-MOFs composites).

In metal–organic skeleton composites, transition metal oxides, such as Fe-MOFs, Co-MOFs, along with various other composite materials, are commonly used. Among these, Fe-MOFs have attracted substantial interest due to their potential applications in catalysis. This fascination arises from the cost-effectiveness, low toxicity, variable valence, biocompatibility, and environmental friendliness associated with the iron (Fe) element [33,34,35]. Fe-MOFs not only inherit several characteristics from conventional MOF materials, such as a large pore volume and a high specific surface area, but they also possess abundant Lewis acid centers, Brønsted acid centers, and unsaturated metal sites, rendering them ideal catalytic active centers [36]. Furthermore, the incorporation of metal ions with redox activity into Fe-MOFs can further enhance their catalytic activity, thanks to the coordination effect. Additionally, the formation of hydrogen bonds between μ_2_-OH or μ_2_-O in the inorganic chains of Fe-MOFs and guest molecules facilitates the efficient removal of pollutants. In comparison to MOFs in the ZIF series and other transition metals like Cr, Ni, Cu, and Co, Fe-MOFs exhibit superior stability, environmental friendliness, and affordability. Prominent examples of Fe-MOFs include MIL-53(Fe), MIL-100(Fe), MIL-88A/B(Fe), MIL-101(Fe), and various functionalized MOFs, often featuring ligands such as Schiff base and pyrazine. As shown in Figure 1, Fe-MOFs are widely used in the study of water pollutants and gaseous pollutants. Their remarkable ability to separate charge carriers contributes to outstanding light absorption and photovoltaic properties. For instance, the Fe_3_-μ_3_-O node-centered cluster structure in the MIL series of Fe-MOFs exhibits a low band gap (Eg) (2.01 eV for MIL-100 (Fe) and 2.62 eV for MIL-53 (Fe)), making it susceptible to the excitation of visible light and the generation of photogenerated electrons–holes. Consequently, Fe-MOFs are widely used in photocatalytic treatment applications [37,38,39,40,41,42]. In addition, it is worth highlighting that Fe-MOFs possess an exceptionally large specific surface area and porosity [43]. When coupled with their ligand-unsaturated sites, such as Lewis acid sites within the structure, this attribute leads to enhanced interactions with guest molecules and reaction intermediates, making them exceptionally well-suited for photocatalytic applications. Li et al. [44] covalently modified NH_2_-MIL-101 (Fe) with 2-anthraquinone sulfonic acid (AQS) and used it as a redox mediator to augment the degradation of bisphenol A through persulfate activation. The result showed that AQS-NH_2_-MIL-101 (Fe) led to an impressive 97.7% degradation of BPA.

This review primarily focuses on the utilization of Fe-based MOFs in the photocatalytic adsorptive removal of harmful water pollutants and gaseous substances commonly found in the environment. The principles governing the removal of water pollutants and large gaseous substances with Fe-MOFs are summarized and discussed. The main reaction mechanism is shown in Figure 1. In addition, their strengths and weaknesses in the context of gaseous pollutant removal are elucidated, while the current research status of Fe-MOFs in the realm of water pollution and air substance photo-treatment is summarized. 

## 2. The Application of Fe-MOFs in Photocatalytic Removal of Organic Pollutants

Organic dyes represent a prominent category of pollutants frequently encountered in dyeing and finishing wastewater. These pollutants, most notably aromatic ring structures like rhodamine B, methyl orange, methylene blue, and malachite green, pose a formidable challenge due to their resistance to complete degradation and removal through conventional treatments [45,46]. Furthermore, the environmental presence of antibiotics, including tetracycline, ciprofloxacin, and chlortetracycline, has garnered significant attention as a major concern. These antibiotic contaminants, resulting from intensive usage and occasional misuse, have led to substantial antibiotic residues in aquatic environments, creating an alarming antibiotic selection pressure. For example, antibiotics are reported to cause the development of resistance in aquatic microorganisms, a phenomenon with direct implications for human health (e.g., superbugs, etc.). Increased resistance escalates infection rates and mortality, posing a serious threat to both human well-being and sustainable development. In addition, commonly used nonsteroidal anti-inflammatory drugs (NSAIDs), such as ibuprofen (IBP), are associated with genotoxicity, aquatic environmental toxicity, and the potential for endocrine disruption in organisms. Organophosphorus pesticides (OPPs), by irreversibly inhibiting important enzymes such as acetylcholinesterase (AChE) in the body, present another environmental challenge. Meanwhile, the plastic monomer and plasticizer bisphenol A (BPA) have been linked to increased macrophage activity, reduced antibody production, and immune system disorders, among other adverse effects [47,48]. In light of these pressing concerns, there is a compelling imperative to explore green methods for the removal of organic pollutants. Photocatalytic degradation emerges as a highly promising approach. Fe-MOFs have played a central role in advancing this field, with notable materials, including MIL-53(Fe), MIL-68(Fe), MIL-88A(Fe), MIL-100(Fe), MIL-101(Fe), the ZIF series, and their composites, forming the mainstay of research in the photodegradation of organic pollutants [49,50,51,52,53,54,55,56].

### 2.1. Dyestuffs Treatment

To harness a broader spectrum of light for the effective utilization of Fe-MOFs, the incorporation of more metal active sites is essential to activate reactants. Designing catalysts with an increased number of active sites stands out as an effective strategy for enhancing photocatalytic performance [57,58,59]. Jin et al. [60] devised a novel dual-ligand Fe-MOF using dielectric barrier discharge (DBD). In this innovative approach, the N ligand served to regulate the active site, while the addition of N enhanced conductivity and provided additional sites for anchoring Fe. This ingenious design ultimately led to the highly efficient degradation of methyl orange. Furthermore, D-Fe-MOFs (dual-ligand Fe-based MOFs) with a greater specific surface area and pore volume were synthesized using DBD [61]. Under simulated sunlight, these materials achieved an impressive 97% degradation of MO (20 mg/L) within 48 min. It was also found that O_2_^−^, ·OH, and h^+^ played a crucial role in the system. During the degradation of MO, the photocatalytic efficiency witnessed a notable decline upon the addition of AO and BQ scavengers, underscoring the significance of holes (h^+^) and superoxide radical anions (O_2_^−^) as the main active species in the photodegradation of methyl orange dye. In contrast, the addition of TBA had a negligible impact on the photodegradation of methyl orange, implying that ·OH played a relatively minor role in the process. Additionally, it was observed that the molar ratios of different organic ligands had exerted a profound influence on the photocatalytic performance of the catalysts. The formation of these ligands resulted in an increased oxygen vacancy and Fe^2+^ content, which in turn facilitated the transport of photogenerated electrons and holes, thus promoting the degradation of MO. Wang et al. [62] employed the BDB plasma method to prepare a composite material consisting of Fe-MOFs@Fe_2_O_3_ using waste PET. Remarkably, this composite material achieved an outstanding degradation rate of 99.3% for malachite green (MG) within 30 min. This innovative process not only demonstrated high-performance MG removal but also contributed to PET plastics recycling.

**Figure 2:**
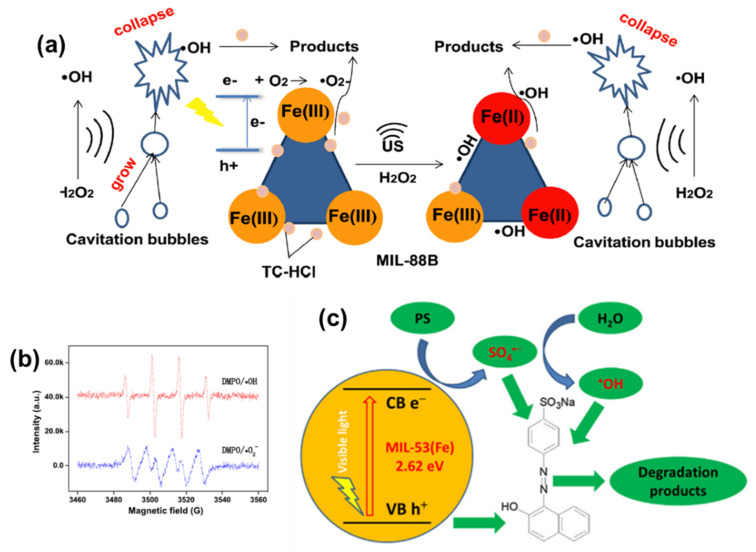
(**a**) Schematic diagram of the proposed mechanism of US/Fenton system for TC-HCl removal; (**b**) EPR spectra of DMPO/·OH and DMPO/·O_2_^−^. Reprinted with permission from Ref. [57]. Copyright 2021, Elsevier. (**c**) Plausible mechanism of photocatalytic degradation of AO7 in the MIL-53(Fe)/PS/Vis process. Reprinted with permission from Ref. [58]. Copyright 2017, Elsevier.

Doping and the formation of complexes represent common strategies employed to improve the photocatalytic performance of Fe-MOFs [63,64]. Metal doping serves to suppress electron–hole recombination to the fullest extent, thereby amplifying the photocatalytic activity of Fe-MOFs. Furthermore, the creation of heterojunction through composites with semiconductor materials extends the spectral response range of Fe-MOFs while also impeding the recombination of photogenerated carriers, prolonging their lifetimes, and consequently enhancing their photocatalytic activity. A significant synergistic effect between sonication and the Fenton reaction was elucidated by Geng [65] et al. They achieved high-performance removal of tetracycline hydrochloride using Fe-MOFs synthesized via an ultrasound/H_2_O_2_/MOF system (Figure 2a,b). The best catalytic performance was observed with MIL-88B, primarily attributable to its higher number of Lewis acid sites. It was experimentally confirmed that ·OH played a pivotal role in the removal of tetracycline hydrochloride, as indicated by EPR testing and quenching experiments. An important synergistic effect between ultrasound and the Fenton reaction was unveiled through a power-factor assessment of the ultrasound. During ultrasound irradiation, microcurrents are generated, promoting mixing at the solid–liquid interface. This mixing enhances the cleansing of the catalyst’s surface, exposing more active sites and accelerating the reaction between H_2_O_2_ and MIL-88B, thereby facilitating the degradation of contaminants. The main reaction pathway in the entire system involves the adsorption and degradation of H_2_O_2_ by unsaturated iron sites on Fe-MOFs. Cao et al. [66] introduced PS into the MIL-53(Fe)/LED visible light photocatalytic degradation system for organic pollutants. PS was employed to facilitate the separation of photogenerated electrons and holes, thereby enhancing the photocatalytic activity of MIL-53(Fe). Under the same experimental conditions, the degradation rate of Acid Orange 7 in the MIL-53(Fe)/PS/LED system reached an impressive 100%, in stark contrast to the MIL-53(Fe)/LED system, which achieved only 24% degradation. This significant enhancement was attributed to PS’s capacity to increase the generation of ·OH in the MIL-53(Fe)/LED system. Photoluminescence spectroscopy (PL) and electron cis-resonance (EPR) analyses confirmed the ability of PS to accept photogenerated electrons, thus promoting the degradation of the dye Acid Orange 7 (Figure 2c). Tran et al. [67], among others, achieved efficient photocatalytic degradation of RhB using bimetallic Mn/Fe-MOF materials obtained by doping Mn^2+^ ions. The activity of these bimetallic centers played a crucial role in shaping unique structures and compositions, ultimately enhancing their photocatalytic ability. Through interfacial conjugation engineering, TiO_2_@NH_2_-MIL-101(Fe) was successfully synthesized to enhance the electron transfer capacity from MOFs to TiO_2_ and induce the band-tail state to obtain a narrower energy band for the composite catalyst. This innovation led to the achievement of high-performance photocatalytic degradation of methylene blue [68]. Brahmi et al. [69] devised MIL-100 (Fe)/polymer and MIL-88A (Fe)/polymer composites using a photopolymerization process, successfully achieving efficient degradation of acidic black pollutants. Tran et al. [70] synthesized M/Fe-MOF (M = Co, Cu, and Mg) materials through bimetallic modification, leading to the efficient removal of rhodamine B. The M/Fe-MOF materials were also modified with M, Cu, and Mg. It was confirmed that after M modification, all metal ions were inserted inside the structure of Fe-MOF material without disturbing their crystal structure. Mousavi et al. [71] successfully synthesized α-Fe_2_O_3_@C@SiO_2_/TiO_2_ with carboxylic acid surface functional groups (α-Fe_2_O_3_@C-COOH) via a two-step dry process. This innovative material demonstrated high-performance removal of dye RY145 while maintaining good recyclability. Zhao et al. [72] found that the presence of amino groups enhances the adsorption capacity of NH_2_-MILs. This enhanced adsorption capability arises from hydrogen bonding interactions and π–π stacking between the amino groups of the dye molecules and the amino-functionalized MOFs. Gong et al. [73] synthesized a core-shell structure, Fe_3_O_4_@GO@MIL-100 (Fe), achieving high-performance degradation of 2,4 dichlorophenol with excellent stability, as well as exo-magnetic field recovery of the catalyst. 

Fe-MOFs also play a key role in the dye-sensitized hydrogen production system, where organic dyes function as photosensitizers, expanding the light response range and facilitating the photocatalytic decomposition of water for hydrogen production within visible light. Li et al. [74] used dye sensitization to enable photocatalytic hydrogen production from Fe-MOFs. Their finding indicated that dye sensitization effectively injects high-energy electrons into Fe-MOFs, enhancing their Fermi energy levels under light. This process helps overcome the surface overpotential for H_2_ production from water. Zhang et al. [75] utilized the coordination and valence mixing of heteroatoms in iron clusters to modulate the electronic energy band structure and charge transfer ability of metal–organic frameworks. They achieved a remarkable twenty-fivefold increase in the hydrogen production rate compared to a pristine Fe-MOF by incorporating Pt as a co-catalyst. ZnIn_2_S_4_@NH_2_-MIL-53(Fe/Co0.75), prepared by Dai et al. [76], achieved an impressive hydrogen production rate of 16,1724.8 μmol/g over a 6 h period. Li et al. [77] attained exceptional photocatalytic hydrogen evolution performance in a dye-sensitized system by anchoring earth-rich copper species to NH_2_-MIL-101 (Fe). The activity achieved, at 5770.96 μmol/g·h, was attributed to a transient Cu^II^/Cu^I^ center, rendering it a noble metal-free synergistic catalyst.

### 2.2. Antibiotics Removal

**Figure 3:**
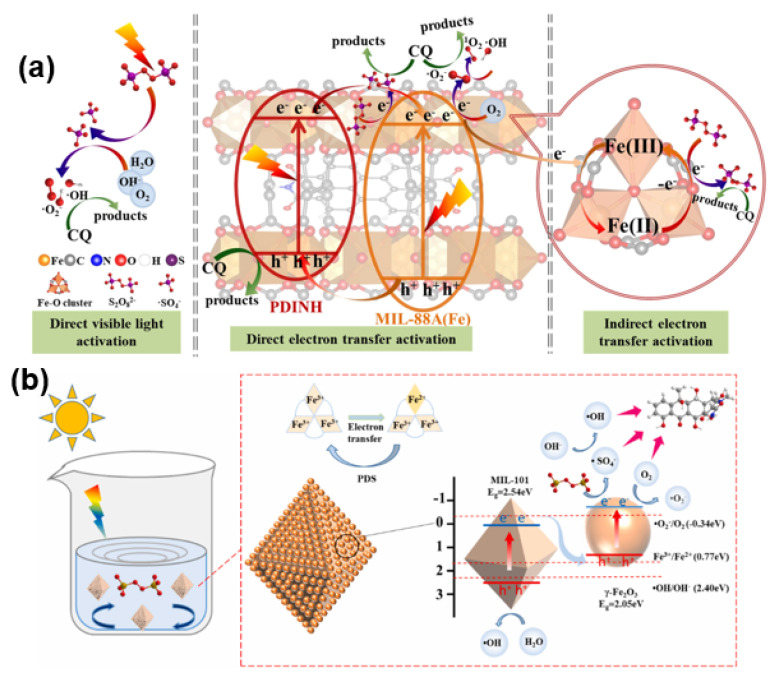
(**a**) Illustration of plausible mechanism of photocatalysis-activated SR-AOP oxidation of CQ over P25M175 under LED visible light. Reprinted with permission from Ref. [80]. Copyright 2021, Elsevier. (**b**) Schematic diagram of the proposed photocatalytic mechanism of OTC degradation in γMF-10/light/PDS. Reprinted with permission from Ref. [82]. Copyright 2022, Elsevier.

He et al. [78] successfully synthesized Fe_3_O_4_@MIL-100 (Fe) hetero-complexes using an in situ growth method, achieving an increase in the removal efficiency of levofloxacin from spiked wastewater from 77.9% to 85.5%. Moreover, the catalyst could be magnetically recycled, thus reducing further environmental pollution. Fu et al. [79] developed the novel MIL-101(Fe)-1-4((ethyl)phenyl)urea (MIL-101(Fe)-EPU) by grafting a phenylethyl side chain onto NH2-MIL-101(Fe). This modification imparted superior hydrophobicity and water stability to the material, resulting in high degradation efficiency for tetrabromobisphenol A. The mechanism was attributed to the oxidation of hydroxyl groups, enabling the degradation of pollutants. Yi et al. [80] achieved excellent degradation of chloroquine phosphate using PDINH/ MIL-88A (Fe) composites prepared through a ball milling strategy. The proposed mechanism is shown in Figure 3a. Yan et al. [81] synthesized CNT@MIL-101 (Fe) with outstanding visible light absorption, enabling efficient removal of ciprofloxacin under visible light irradiation. Du et al. [82] accomplished the efficient removal of oxytetracycline (OTC) utilizing a magnetic nanocomposite, MIL-101(Fe)/γ-Fe_2_O_3_, synthesized by a hydrothermal method. This catalyst could be recovered magnetically, enhancing its recyclability (Figure 3b). By modifying the ligand structure and fine-tuning the coordination of 2-MI to control the crystal size and morphology of Fe-MOFs, Cheng [83] et al. achieved the first one-pot hydrogenation and N-alkylation reaction of benzopropionitrile with alcohols, demonstrating excellent performance. Moreover, visible light irradiation was found to significantly enhance the activation efficiency compared to heating through in situ infrared. Liu et al. [84] obtained a magnetic Fe_3_O_4_@MIL-53(Fe) photocatalyst by calcining pristine MIL-53(Fe). This material exhibited high-performance degradation of ibuprofen under visible light irradiation.

### 2.3. Photo-Fenton System

**Figure 4:**
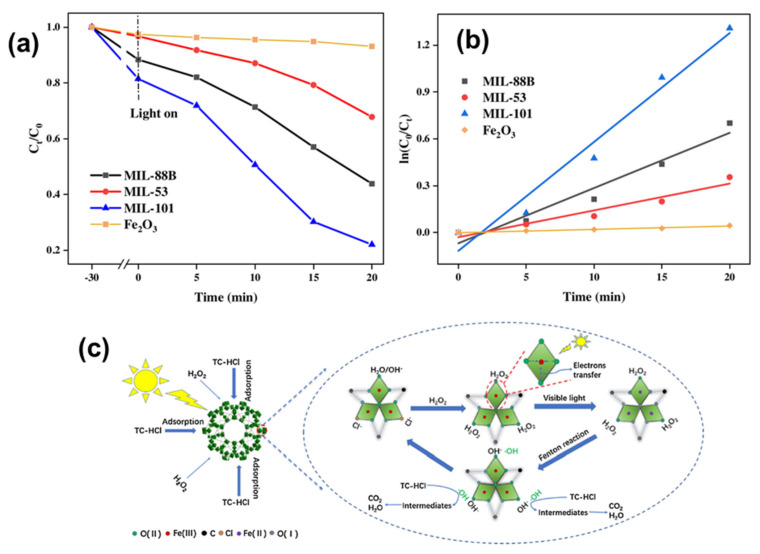
(**a**,**b**) Photo-Fenton degradation of TC-HCl and the corresponding kinetics with various Fenton-like catalysts. Experimental conditions: [TC-HCl] = 50 mg/L, [Catalyst] = 0.10 g/L, [H_2_O_2_] = 10 mL/L, pH = 4.1, T = 4.1, T = 287 K, visible light. (**c**) Illustration of the proposed reaction mechanism for TC-HCl removal in MIL-101/H_2_O_2_/visible light system. Reprinted with permission from Ref. [91]. Copyright 2020, Elsevier.

Metal–organic frameworks (MOFs), particularly iron-based MOFs (Fe-MOFs), are emerging as promising Fenton-like catalysts due to their well-developed pores and available active sites [85,86,87]. However, the strong coordination between metal ions and organic ligands often reduces the exposure of active sites in most iron-based MOFs, limiting Lewis acid site availability and thus hampering the activation efficiency of H_2_O_2_. In addition, the lower cycling efficiency of Fe^3+^/Fe^2+^ further limits the activation efficiency of H_2_O_2_. Therefore, there is a significant focus on improving the cycling efficiency of Fe^2+^/Fe^3+^. Horiuchi et al. [88] discovered that MIL-101 (Fe) promotes photocatalytic water oxidation under visible light irradiation, producing oxygen from a silver nitrate solution. The finely dispersed Fe-O clusters had a very effective positive impact on the reaction. Huang et al. [89] synthesized a surface-coated Fe-MOF with recrystallized PDI (perylene-3,4,9,10-tetracarboxylic diimide), where the Fe-MOF in the northern part of the coating remained intact due to the stabilized H-type π–π stacking of the PDI under acidic conditions. The resulting Fe-MOF@PDI photocatalyst exhibited increased acid resistance, resulting in enhanced Fenton activity and stability. A series of modulations of α-Fe_2_O_3_ at the nanoscale using surface engineering was carried out by Xu et al. [90], resulting in catalysts with various shapes, such as spherical, octahedral, spindle, rod-shaped, and controllable oxygen vacancies (Ovs) of α-Fe_2_O_3-x_. Based on the photocatalytic results, rod-shaped α-Fe_2_O_3-x_ exhibited the best performance in the complete degradation of methylene blue (MB). This performance was attributed to the synergistic effect of a large specific surface area, a high concentration of Fe^2+^, and Ovs. Using Fe-MOFs, Wu et al. [91] demonstrated, for the first time, that visible light can accelerate the Fe^2+^/Fe^3+^ cycling in a photo-Fenton system based on Fe-O clusters within the framework. This acceleration promoted the degradation of tetracycline hydrochloride. Utilizing a solid acid catalyst, MIL-101 effectively extended the pH range up to 10.2 while also demonstrating good reusability and stability (Figure 4a,b). The proposed mechanism is presented in Figure 4c.

**Figure 5:**
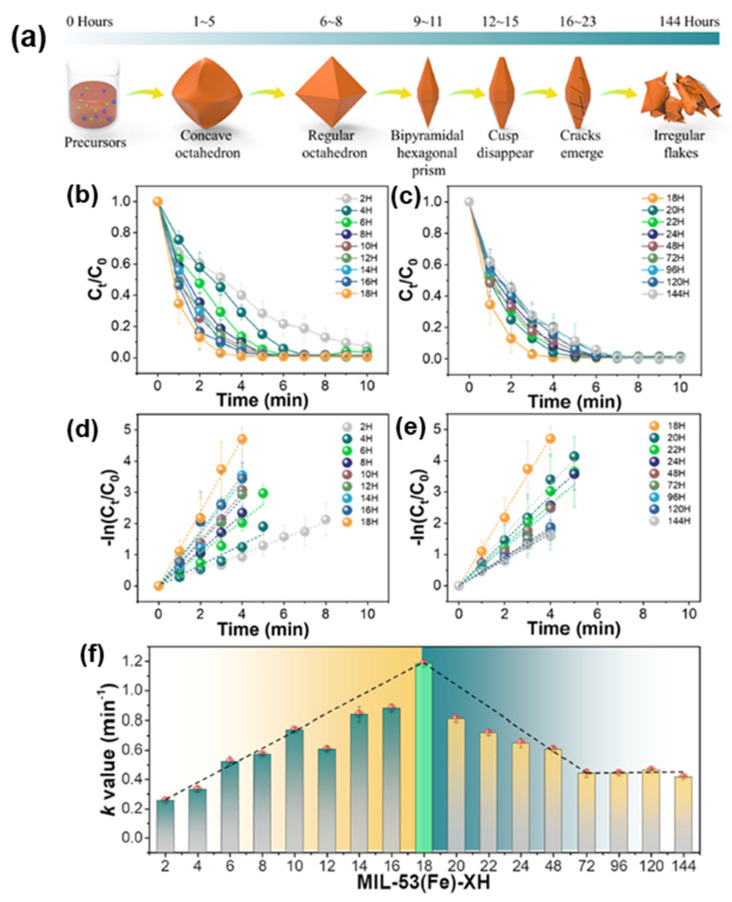
(**a**) Schematic illustration of the morphology evolution progress of MIL-53(Fe)s as crystallization time increases. (**b**,**c**) Evolution of normalized RhB concentration versus reaction time as a function of the screened MIL-53(Fe)-XH (X = 2, 4, 6, 8, 10, 12, 14, 16, 18, 20, 22, 24, 48, 96, 120, and 144) over HCO process. (**d**,**e**) Fitting results of RhB destruction by a series of MIL-53(Fe)s using a pseudo-first-order kinetic model. (**f**) Pseudo-first-order rate constants for probe reactions and the volcano plot of probe destruction rate constants over the solvothermal reaction time for MIL-53(Fe)s synthesis. Experimental conditions: [RhB]_0_ = 100 mg/L, [caralyst] = 0.1 g/L, [O_3_]_in_ = 30 mg/L, gas flow rate = 60 mL/min, T = 25 °C. Reprinted with permission from Ref. [93]. Copyright 2022, Elsevier.

Guo et al. [92] accomplished the degradation of tetracycline hydrochloride (HT-HCl) under visible light by hydrothermally synthesizing a series of mixed-valence coordinated unsaturated metal sites (CUSs) with varying Fe^2+^/Fe^3+^ ratios in MIL-100 (Fe). This is due to the fact that visible light irradiation accelerated the cycling of Fe^2+^/Fe^3+^, resulting in enhanced catalytic performance. The synergistic relationship between the porous structure of Fe-MOFs and the exposure of active sites for APO catalysis was intensively investigated by Hu et al. [93] (Figure 5a). It was found that the ozone-catalyzed reaction activity of MIL-53(Fe) was regulated by the evolution of crystal structures over time. Six evolutionary steps of MIL-53(Fe) crystals under the action of intramolecular hydrogen bonding were revealed, leading to a volcano plot depicting the relationship between rhodamine degradation-catalyzed ozone reactivity and crystallization time (Figure 5b–f).

**Figure 6:**
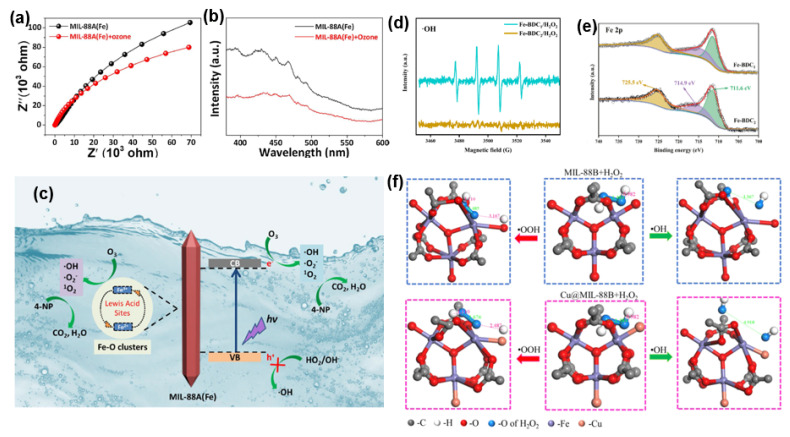
(**a**) EIS Nyquist impedance plots and (**b**) PL spectra of MIL-88A(Fe) in the presence or absence of ozone. (**c**) Proposed PCO mechanism of MIL-88A(Fe) for 4-NP degradation and mineralization. Reprinted with permission from Ref. [95]. Copyright 2019, Elsevier. (**d**) EPR spectra with DMPO (·OH) as trapping agents in different systems. (**e**) Fe 2p spectra of Fe-BDC_1_ and Fe-BDC_2_. Reprinted with permission from Ref. [96]. Copyright 2022, Elsevier. (**f**) Model of H_2_O_2_ dissociation process in MIL-88B and Cu@MIL-88B. Reprinted with permission from Ref. [98]. Copyright 2023, Elsevier.

Yang et al. [94] modified the structure of the Fe-O node in MIL-53(Fe) by substituting Fe^2+^ to obtain a catalyst with mixed valence coexistence. This modification led to excellent catalytic degradation of 4-nitrophenol. The substitution of Fe^2+^ introduced higher active sites than the original Fe^3+^ center (Fe^2+^→Fe^3+^ half-reaction) rapidly activating H_2_O_2_ and efficiently destroying 4-nitrophenol. It was also found that the accelerated cycle of Fe^2+^ and Fe^3+^ coupling promoted increasing ·OH generation and allowed more 4-NP to be degraded. Using Fe-MOF with more Lewis acid sites (LAS), Crittrnden et al. [95] obtained a synergistic effect that enhanced photogenerated carrier separation when ozone served as the electron acceptor. It was also demonstrated that ozone not only reduced the photogenerated carrier recombination but also decomposed on the LAS of MIL-88A (Fe) to generate more reactive oxygen species, further promoting the degradation of 4-nitrophenol (Figure 6a–c). Fe-BDC1, synthesized by Wu et al. [96] under simple stirring conditions, achieved high-performance degradation of Rhodamine B. The degradation of Rhodamine B was achieved by Fe-BDC1. Iron coordination unsaturated sites (Fe CUSs) and ·OH were also identified as active species for efficient degradation of the pollutant (Figure 6d,e). Tang et al. [97] accomplished the degradation of ibuprofen by anchoring ReS_2_ nanoparticles in a heterojunction formed in MIL-88B (Fe) under visible light and persulfate (PS). Song et al. [98] obtained mixed-valent Cu@MIL-88B(Fe) after processing the Fe-oxo node structure in MIL-88B(Fe) by the Cu^+^ species substitution method. Both experimental and computational results showed that Cu^+^ played a shuttling role in promoting the transfer between Fe^2+^/Fe^3+^ and inducing the formation of a large number of stable Fe^2+^ sites, as well as suppressing the leaching of iron ions during the catalytic process. These modifications significantly improved the performance of the Fenton-like reaction (Figure 6f).

### 2.4. Hexavalent Chromium Removal

Cr (VI) is a common highly toxic water pollutant originating from various sources like leather tanning, cooling tower discharge, electroplating, and anodizing baths. It poses harmful effects by penetrating the cell wall, unlike the less toxic Cr (III), which is almost insoluble at neutral pH. Therefore, reducing Cr (VI) to Cr (III) is a key process for the effective removal of Cr (VI) from water and wastewater [99,100]. 

**Figure 7:**
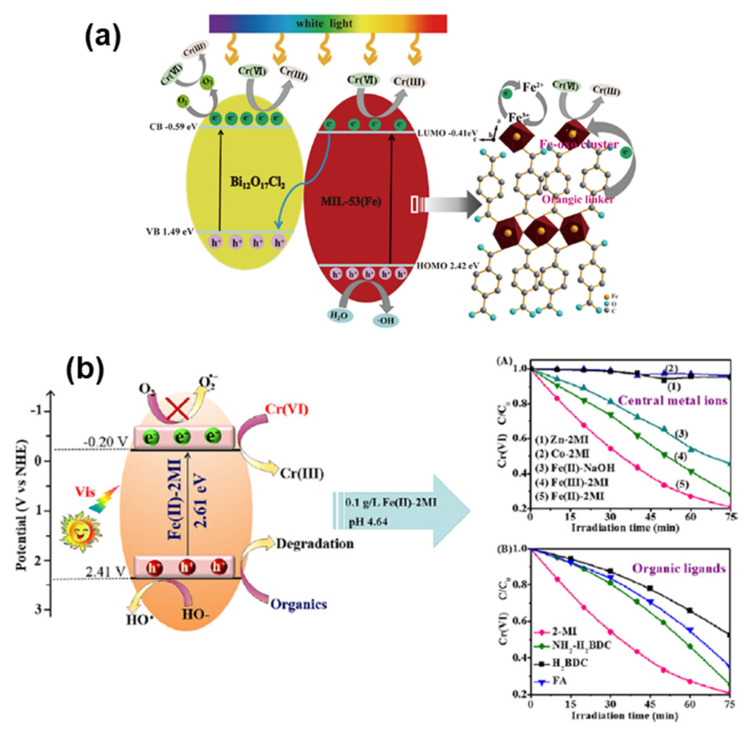
(**a**) A schematic diagram of Ce(VI) photoreduction over the MB100 under white light illumination. Reprinted with permission from Ref. [101]. Copyright 2020, Elsevier. (**b**) Self-assembled and amorphous Fe-2MI displayed fascinating photocatalytic activity for Cr(VI) reduction. Reprinted with permission from Ref. [104]. Copyright 2019, Elsevier.

The reduction of Fe^3+^ to Fe^2+^ in the linker-to-metal clusters of Fe-MOFs is crucial for charge transfer. Wang et al. [101] constructed a series of MIL-53(Fe) and Bi_12_O_17_Cl_2_ (MB_x_) composites by ball milling, demonstrating superior Cr(VI) degradation performance under visible light irradiation. They also confirmed that Fe-μ_3_-oxo clusters in MIL-53(Fe) triggered the Cr(VI) reduction process through linker-to-metal-cluster charge transfer (LCCT), which reduced Fe^3+^ to Fe^2+^, facilitating Cr(VI) removal. Graphite-like phase C_3_N_4_, a metal-free semiconductor material known for its π–π conjugated electronic structure, narrow bandgap (~2.7 eV), excellent chemical stability, good thermal stability, and low cost, is increasingly used for Fe-MOF modification (Figure 7a). Zhang [102] et al. successfully synthesized four novel iron-based metal–organic frameworks (MOFs) through ligand modulation to achieve improved Cr(VI) reduction properties. MTBDC-TPT-Fe exhibited the best performance due to enhanced electron push–pull effects between the iron–oxygen clusters and the organic ligands. The introduction of the -SCH_3_ group enhanced light absorption of the MOFs and provided electrons for the Fe centers, while TPT enhanced the photogenerated charge carriers’ separation and transfer. Wang et al. [103] introduced p-type carbon nitride (CN75) nanoparticles into NH_2_-MIL-53(Fe) through a solvothermal method to form p-n heterojunctions. This promoted charge carrier separation and migration, broadened visible light response, and achieved efficient hexavalent chromium photoreduction. At the same time, the sample also exhibited excellent cyclic stability and structural stability. Gao [104] et al. synthesized Fe-2MI compositions with visible light response using 2-methylimidazole (2MI) as an organic ligand. This led to the formation of complexes with Fe^2+^ and Fe^3+^ where Fe^2+^ formed a more stable complex with a higher coordination ratio with 2MI. Fe^2+^-2MI had suitable conduction and valence band positions for the reduction of Cr (VI) and organic matter oxidation, demonstrated in the mechanism study (Figure 7b). From the right side (A) of Figure 7b, it can be seen that compared to other samples, Fe (II)-2MI exhibited the best activity for Cr (VI) reduction. In the comparative experiment of different organic ligands (2MI, H_2_BDC-NH_2_-H_2_BDC, and FA), it can be seen that Fe (II)-2MI still exhibited the best performance (Figure 7b, right (B)). Meanwhile, He et al. [105] synthesized Fe-BDC/Fe-2MI heterojunctions via a one-pot method. They observed the presence of MIL-88B (Fe) in the Fe-BDC/Fe-2MI complexes, unlike MIL-53 (Fe) in bare Fe-BDCs, indicating the modulating effect of 2MI on the structure of Fe-BDCs.

### 2.5. Other Water Pollutants

In addition to dyes, antibiotics, and heavy metals, Fe-MOF photocatalysts have shown promise in the removal of various other water pollutants. Oladipo et al. [106] achieved the decomposition of two organophosphorus pesticides, methylmalathion (MP) and chlorpyrifos (CP), under sunlight irradiation using AgIO_3_/MIL-53(Fe). The individual degradation rates of CP and MP were 78% and 90%, respectively, under 60 min of sunlight irradiation in both tap water and distilled water. The individual degradation rates of CP and MP in tap water and distilled water were 78% and 90%, respectively, under 60 min sunlight irradiation. Zhong et al. [107] prepared Cu_2_O/MIL(Fe/Cu) composites through an in situ copper bridge strategy to enhance visible light absorption and achieve efficient degradation of thiacloprid (TCL). It was demonstrated that the Cu bridge promoted the charge interfacial transfer and Fe^2+^/Fe^3+^ redox reaction between Cu_2_O and MIL(Fe/Cu). Ahmad et al. [108] successfully synthesized stable mesoporous MIL-100(Fe)-loaded ZnO NS using an in situ self-assembly method. This composite achieved high-performance removal of chlorpyrifos and malathion–methyl. It was also found that the catalytic activity of mesoporous MIL-100(Fe) was significantly enhanced thanks to the mesoporous structure, which improved the diffusion and accessibility of molecules within the MOF channel.

### 2.6. Simultaneous Removal of Multiple Water Pollutants

**Figure 8:**
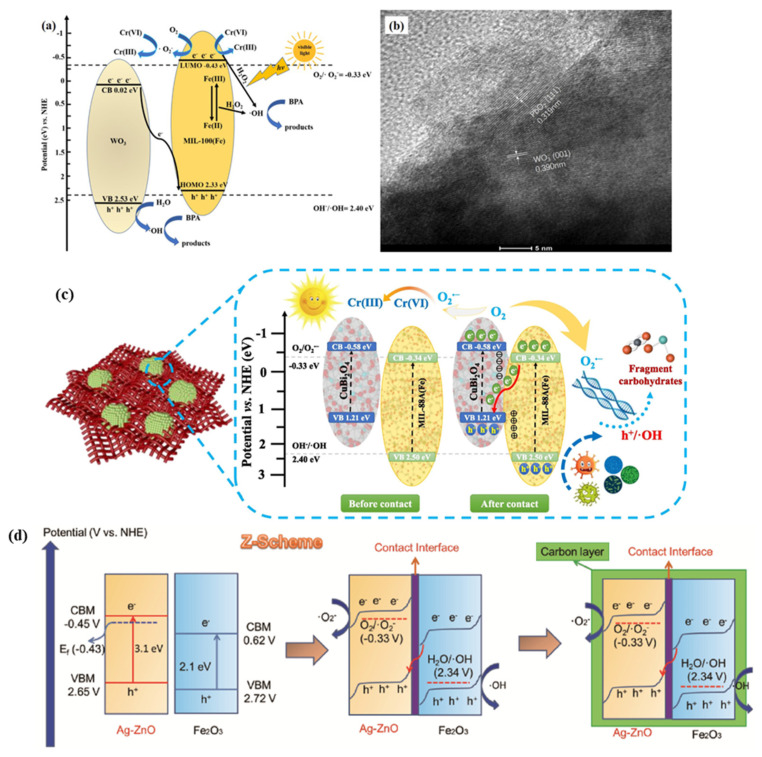
(**a**) The diagram of photocatalytic Cr(VI) sequestration and photo-Fenton BPA decomposition mechanism of M80W120; (**b**) HRTEM images of photo-deposited PbO_2_ over M80W120. Reprinted with permission from Ref. [110]. Copyright 2021, Elsevier. (**c**) Proposed charge transfer mechanism toward Cr(VI) reduction and antibacterial applications by CBO/MIL-88(Fe). Reprinted with permission from Ref. [114]. Copyright 2023, Elsevier. (**d**) Proposed Z-scheme photocatalytic system of FAZ. Reprinted with permission from Ref. [115]. Copyright 2022, American Chemical Society.

In recent years, researchers have been exploring the simultaneous removal of heavy metals and the byproduction of valuable products during the process of removing organic pollutants [109]. Wang’s group prepared Z-Scheme WO_3_/MIL-100(Fe)(M_x_W_y_) composites using a ball milling strategy [110]. This composite exhibited high performance in the removal of both hexavalent chromium and BPA. The successful synthesis of the Z-Scheme structure was confirmed with a series of characterizations, and it was found to contribute positively to the efficient removal of Cr (VI) and BPA (Figure 8a,b).

In addition to introducing an oxygen source with strong electron trapping ability into the photocatalytic system of MOFs, another strategy to enhance the photocatalytic activity of MOFs is to prepare heterostructured materials by compositing with photoresponsive materials (e.g., TiO_2_, g-C_3_N_4_)/strong electron transporting materials (e.g., (oxidized) graphene). Similarly, the role of such strategies ultimately is to promote photogenerated electron and hole separation [32,52,111]. Liu et al. [112] prepared a sandwich-like heterostructure, TiO_2_NS@MIL-100(Fe), by combining MIL-100(Fe) with two-dimensional TiO_2_ nanosheets via a self-assembly method for the photocatalytic degradation of methylene blue. The interface between MIL-100(Fe) and TiO_2_ nanosheets facilitated the rapid photogenerated electron transfer and conversion and enhanced the photocatalytic activity of TiO_2_NS@MIL-I00(Fe). The g-C_3_N_4_/NH_2_-MIL-101 (Fe) prepared via the in situ solvothermal method by Liu et al. [113] achieved efficient photocatalytic removal of Cr (VI) and MO. The pH was found to be crucial for the reduction of Cr (VI). It was also found by infrared spectroscopy that as the content of g-C_3_N_4_ increased, the corresponding absorption became stronger, indicating the successful incorporation of g-C_3_N_4_ into the composite. The Z-Scheme CBO/MIL-88A(Fe)p-n heterojunction constructed by Li et al. [114] achieved nearly 100% removal of hexavalent chromium within 30 min under visible light irradiation. In terms of antimicrobial activity, complete inactivation was also achieved within 60 min. Notably, the leaching of iron ions was minimal after several cycles (Figure 8c).

The heterostructure of Fe_2_O_3_@Ag-ZnO@C of Z-Scheme designed by Li et al. [115] demonstrated efficient degradation of tetracycline and methylene blue in wastewater. Through a combination of experimental and computational results, it was found that the Fe_2_O_3_@ZnO nano-interface and the carbon sheath worked together to enhance the efficiency of photogenerated carrier transfer and absorption capacity. Additionally, the treated wastewater showed good biocompatibility (Figure 8d). Nguyen et al. [116] utilized a microwave-assisted solvothermal method to synthesize a series of M/Fe-MOFs (M = Ni, Mg, and Sn) for the high-performance and sustainable removal of different dyes, including rhodamine B (RhB), crystal violet (CV), methyl orange (MO), and methylene blue (MB).

## 3. The Application of Fe-MOF Photocatalytic Removal and Conversion of Gaseous Substances

The distinctive structural characteristics and presence of unsaturated metal sites in Fe-MOFs have led to numerous applications in the field of the photocatalytic removal of gaseous pollutants. These applications predominantly revolve around the photoreduction of CO_2_, the elimination of NO_x_, nitrogen fixation, and the abatement of VOCs. 

### 3.1. CO_2_ Removal

The imperative to mitigate CO_2_ emissions has emerged as a pressing environmental concern. At the same time, combating climate change through controlling CO_2_ emissions has become an international consensus. CO_2_ capture methods primarily encompass absorption, adsorption, biofixation, membrane separation, and photocatalysis. Among them, photocatalysis has been widely used due to its environmentally friendly and low-carbon attributes. The utilization of MOF materials in photocatalytic CO_2_ removal represents a burgeoning research area in recent years, with Fe-based MOFs occupying an indispensable role [117,118,119,120,121,122,123,124,125,126].

**Figure 9:**
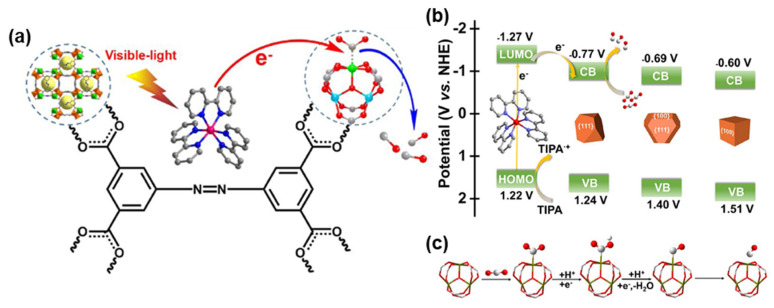
(**a**) A scheme of photocatalytic routes for visible-light-driven CO_2_ reduction in PCN-250-Fe_2_M (or PCN-250-Fe_3_). Reprinted with permission from Ref. [129]. Copyright 2020, Elsevier. (**b**) Illustration of the Band Structure and Photoinduced Electron Transfer Progress. (**c**) Illustration of the Intermediate during CO_2_-to-CO Conversion over Fe_3_O Clusters. Reprinted with permission from Ref. [130]. Copyright 2022, American Chemical Society.

Subsequently, a series of NH_2_-MIL-101 (Fe) catalysts prepared by Dao et al. [127] demonstrated an efficient and highly selective reduction in CO_2_ at the gas–solid interface. NH_2_-MIL-101(Fe) obtained by Wang et al. [128] via growing varying metal ions in situ onto covalent frameworks containing triazines exhibited remarkable CO_2_ reduction to CO and CH_4_. Dong et al. [129] synthesized MOF-PCN-250-Fe2M, featuring Fe^2+^/Fe^3+^ metal cluster nodes and open metal sites, and further enhanced its CO_2_ photoreduction performance through modulation of MII metal ions in the metal clusters (M = Mn, Zn, Ni, Co) (Figure 9a). Zhang et al. [130] synthesized Fe-soc-MOF (Fe-soc-O) with eight {111} crystalline facets exposed and Fe-soc-MOF (Fe-soc-M) with eight {111} crystalline facets and six {100} crystalline facets, achieving high-performance CO_2_ photoreduction. Notably, the surface-active sites of Fe-soc-MOF were found to be closely related to the crystal faces, with Fe-soc-O exhibiting the highest CO generation and Fe-soc-M displaying superior CO selectivity (Figure 9b,c).

### 3.2. NO_x_ Removal and Nitrogen Fixation

**Figure 10:**
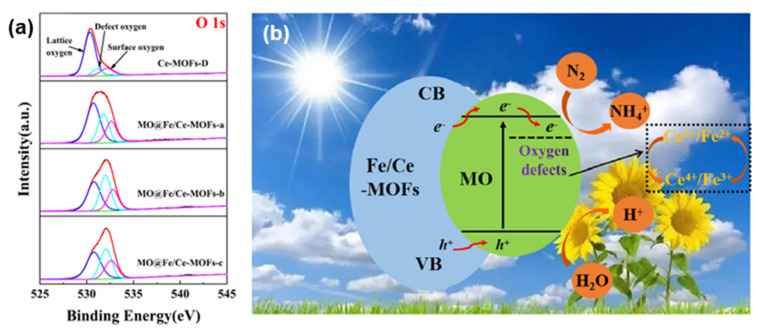
(**a**) O 1s spectra of Ce-MOFs-D and MO@Fe/Ce-MOFs. (**b**) Possible mechanism of photocatalytic nitrogen fixation. Reprinted with permission from Ref. [137]. Copyright 2023, Elsevier.

Nitrogen oxides, inherently toxic and detrimental to the ozone layer, contribute to the formation of photochemical smog, acid deposition, and eutrophication of water bodies. Excessive levels also pose direct threats to human respiratory health, presenting a significant risk to humanity’s well-being. While widely used catalysts like V_2_O_5_-WO_3_ (MoO_3_)/TiO_2_ are effective, the presence of toxic vanadium can compromise their selectivity for nitrogen, leading to the production of N_2_O or NO at high temperatures [131,132,133]. It has been shown that Fe-MOFs can form a large number of coordination unsaturated metal centers (CUSs) serving as Lewis acid centers in the NH_3_-SCR catalytic system [134]. N_2_ desorbs into the gas phase, forming Brønsted acid sites on the backbone, where NH_3_ adsorption is more favorable, facilitating the reaction. Therefore, Fe-MOFs find extensive use in this industry. Li et al. [132] observed exceptional photocatalytic nitrogen fixation activity in a series of Fe-based MOFs (MIL-101 (Fe), MIL-100 (Fe), and MIL-88 (Fe)). Photocatalytic nitrogen fixation activity was compared between MIL-101 (Fe) and MIL-101 (Cr), with the former exhibiting an activity of 50.355 μmol/h, while the latter remained inactive. This led to the conclusion that the source of nitrogen fixation activity was the iron catalytic center. Given the similarity of the outer electronic structures of Cr and Fe, it is hypothesized that the two extra multi-site electrons of elemental Fe are the fundamental reason for the excellent photocatalytic nitrogen fixation activity. Nguyen et al. [135] synthesized MIL-101 (Fe) using a facile microwave solvothermal method and employed it as a photocatalyst for NO_x_ degradation. The results showed that under the optimal reaction conditions (alumina matrix, initial NO concentration of 350 ppb, and relative humidity of 40–50%), photocatalytic degradation of NO_x_ under sunlight irradiation of 0.1 g of the material reached an efficiency of up to 77%, offering optimal conditions for efficient photocatalytic degradation of NO_x_. MIL-101, when exposed to appropriate light, generates charges at the Fe_3_-μ_3_-OXO cluster or BDC (benzene dicarboxylic acid ligand) junction, which then oxidize and reduce H_2_O and O_2_ to ·OH and ·O_2_^−^ for catalytic removal of pollutants. During irradiation, the competition between the guest molecules (H_2_O, NO, and O_2_) and the Fe active center significantly impacts denitrification performance. Chen et al. [136] utilized the H_4_abtc ligand attached to an iron cluster to create the stable metal–organic framework Fe-abtc, achieving nitrogen fixation under visible light. The stable structure formed by linking the tetracarboxylic acid to the metal cluster and the presence of N@N in the ligand is crucial to the sustainability of the photocatalytic reaction. Huang et al. [137] greatly promoted the photocatalytic nitrogen fixation reaction using oxygen-deficient MO@Fe/Ce-MOFs synthesized via dielectric barrier discharge (DBD) plasma. Their work provided a detailed description of the critical role played by oxygen defects in nitrogen fixation reactions (Figure 10a,b). Li et al. [132] demonstrated outstanding photocatalytic nitrogen fixation activity in a series of Fe-MOFs, highlighting the iron catalytic center and its two extra divalent electrons as the key factors behind this exceptional performance.

### 3.3. VOCs Removal

**Figure 11:**
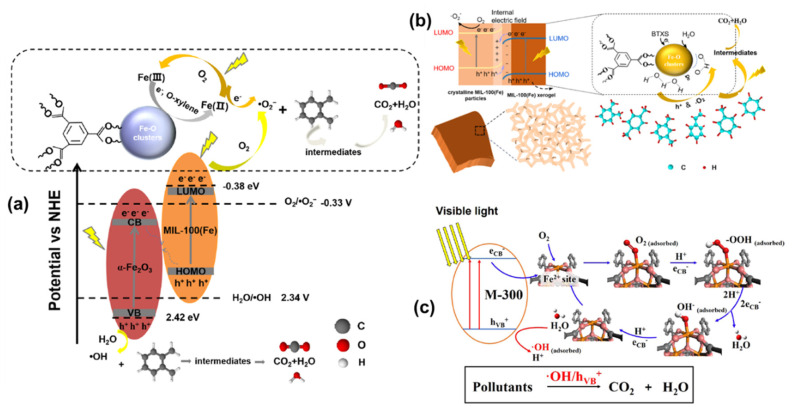
(**a**) Direct Z-scheme mechanism in MIL-100(Fe)/α-Fe_2_O_3_ hybrid. Reprinted with permission from Ref. [140]. Copyright 2021, Elsevier. (**b**) The mechanism of photocatalytic degradation of BTXS on the MIL-100(Fe) MOF/MOX homojunctions. Reprinted with permission from Ref. [141]. Copyright 2022, Elsevier. (**c**) The mechanism for photocatalytic oxidation on M-300 catalyst under visible light. Reprinted with permission from Ref. [142]. Copyright 2023, Elsevier.

VOCs are known for their strong irritant and toxic properties, with some even being teratogenic and carcinogenic. Common treatment technologies for VOCs include adsorption, absorption, condensation, membrane separation, catalytic combustion, thermal incineration, biodegradation, photocatalytic methods, and plasma technology. MOFs, characterized by their substantial specific surface area and strong catalytic activity, hold significant promise for VOC treatment applications. They have been extensively studied and applied as catalysts for photocatalytic degradation of VOCs [120], including applications involving Fe-based MOFs. Li et al. [138] proposed an efficient and stable Fe-based metal-organic skeleton (Fe-MOF) in which adsorption and photodegradation regeneration alternated to achieve repetitive and efficient removal of VOCs. The Fe-MOF could be regenerated by converting adsorbed gases into CO_2_ under 1 solar (100 mw/cm^2^) irradiation, demonstrating excellent durability over 100 cycles of repeated adsorption and degradation. Jiang et al. [139] synthesized Pd/Fe MOFs nanocomposites through coordination engineering, achieving the conversion of various aldehydes, alcohols, and toluene into amide compounds and amines using only flue gas (or air) as an oxidant, with water as a byproduct. Chen et al. [140] synthesized MIL-100 (Fe) by adjusting the coordination of Fe^3+^ ions/α-Fe_2_O_3_, achieving efficient removal of ortho xylene. This study also confirms the critical role played by the reversible conversion of Fe^3+^ and Fe^2+^ in the oxidation of ortho-xylene and the effective generation of active radicals (Figure 11a). Subsequently, the team utilized MIL-100 (Fe)/MOX synjunction to efficiently capture BTXS (benzene, toluene, xylene, and styrene) using coordination of unsaturated acidic Fe_3_-O sites [141], participating in the PCO process through the conversion between Fe^3+^ and Fe^2+^ (Figure 11b). Qin et al. [142] prepared a Fe MOF derivative (M-300) using MIL-100 (Fe) as the precursor, exhibiting excellent performance in degrading VOCs and eliminating Escherichia coli under visible light. At the same time, it achieved high-performance visible light degradation of acetaldehyde. This was attributed to the exposure of unsaturated Fe^2+^ active sites, which promoted the transfer of photogenerated carriers and redox processes, thereby improving the overall photocatalytic performance of the system (Figure 11c).

The synthesis methods of relevant catalysts and the removal performance of pollutants are shown in Table 1.

## 4. Strengths and Challenges

In the field of photocatalysis, most conventional catalysts consist of semiconductor materials. However, Fe-MOFs offer significant advantages by substantially enhancing the porosity of the materials and finely regulating inherent Lewis acidity through the incorporation of Fe as a node. Moreover, the combination of Fe ion nodes with organic ligands as bridge compositions renders Fe-MOFs a promising catalyst for various applications. Fe-MOFs can also be effectively integrated with functional materials, such as metal oxides, carbon-based materials, and metal nanoparticles, to construct new composites. 

The advantages of Fe-MOFs can be summarized as follows:

(1) Fe-MOFs exhibit a wide spectral response, enabling them to catalyze chemical reactions within the visible light spectrum. This characteristic enhances their applicability. Fe-MOFs find applications in photocatalytic processes focused on degrading organic pollutants, converting CO_2_ through photocatalysis, nitrogen fixation, and degrading of gaseous organic pollutants.
(2) Featuring uniform, stable, and infinitely elongated structural composition units, Fe-MOFs allow for precise structural regulation of composite materials by adjusting the structural composition of MOFs.
(3) Fe-MOFs possess high porosity, an intricate pore structure, and an extremely large internal specific surface area. Additionally, the pore size is controllable, providing ideal conditions for introducing other precursors into the pore structure.
(4) Fe-MOFs exhibit a soft nature and can be modified by altering external conditions during the synthesis process. Factors such as temperature, pressure, acidity, and alkalinity can be adjusted to tailor the material’s properties as needed.

However, to advance the application of Fe-MOFs in catalytic water pollution purification and atmospheric substance treatment, several key improvements are necessary:

(1) While Fe-MOF photocatalytic materials have found applications in water pollutants and atmospheric treatment, the current study focus has primarily been on novel MOF photocatalysts and the effect of ligand variations on the photocatalytic activity of MOFs. There exists a notable gap in systematic research concerning the photocatalytic mechanism of MOF materials and the relationship between photocatalytic performance and material structure. Future research efforts should prioritize strengthening and enhancing our understanding in these areas.
(2) Fe-MOFs encounter challenges related to stability, high production cost, complex preparation processes, and limited photocatalytic activity. While some auxiliary methods have shown promise in shortening the synthesis time of Fe-MOF photocatalytic materials and improving their catalytic activity, many of these studies remain in the experimental stage. It is crucial to further develop and scale up these techniques to facilitate their industrial application.
(3) Current research has placed relatively less emphasis on the effective methods of separating used catalysts from the reactants. To enhance the practical industrial application of these materials, it is essential to explore approaches such as imparting magnetic properties to the materials or converting them into loaded catalysts. How to combine the good photocatalytic degradation function with recyclability and low cost remains a challenging yet crucial issue to address.
(4) Utilizing advanced fine structure analysis and density functional theory calculations, researchers can delve deeper into the conformational relationship between the microstructure and macroscopic performance of the catalysts. This approach allows for a more profound understanding of the interfacial electron transfer mechanism, guiding the design and regulation of catalysts with improved catalytic activity.
(5) The synergistic removal of various pollutants in complex environment settings should be further emphasized to maximize the excellent catalytic performance of Fe-MOF composite catalysts when addressing real-world challenges.

## 5. Conclusions and Outlook

In summary, this review highlights recent advancements in the utilization of Fe-MOFs-based catalysts for highly efficient photocatalytic degradation of water and atmospheric pollutants. It has been emphasized throughout this review that functionalized Fe-based MOFs, as well as their derivatives and complexes, have successfully addressed challenges related to photogenerated carrier susceptibility to complexation, Fe^2+^/Fe^3+^ transfer, and insufficient exposure of the active site compared to pristine Fe-based MOFs. These innovations have significantly expanded the range of applications for Fe-MOFs and have shown promising results in both water pollution and air substances remediation. This review provides a comprehensive overview of the current state of research on Fe-MOFs and their composites, highlighting advanced materials used in photocatalytic technologies aimed at achieving the complete removal and mineralization of pollutants present in water and the atmosphere.

Despite the existing challenges, Fe-MOFs hold a promising outlook in the removal of environmental pollutants. With the continued dedication of scholars from various fields, it is anticipated that Fe-MOFs will eventually find practical applications in water and atmospheric purification. In the future, the development trajectory of Fe-MOF photocatalytic materials can be explored through efforts aimed at reducing production time, achieving large-scale production, reducing the cost, simplifying the preparation processes, and enhancing the catalytic activity. As science and technology continue to advance, future Fe-MOF materials will usher in a more vigorous development in the field of photocatalysis.

## Figures and Tables

**Table 1:** The degradation performance of Fe-based MOFs and their composites.

Catalysts	Synthesis Method	Target Substances	Concentration(mg·L^−1^)/Volume (mL)	Catalyst (g/L)/ (g)	Reaction Time (min)	Efficiency (%)/Evolution	Light Source	Ref.
N-Fe-MOFs	DBD plasma	MO	20	0.80	48	97.00	500 W xenon lamp	[60]
D-Fe-MOFs	DBD plasma	MO	20	0.80	48	97.00	500 W xenon lamp	[61]
Fe-MOFs@Fe_2_O_3_	DBD plasma	MG	15	0.30	20	99.30	500 W xenon lamp	[62]
MIL-88B(Fe)	Solvothermal	TC-HCl	10	0.20	7	83.30	/	[65]
MIL-53(Fe)	Solvothermal	AO7	0.05 mM	0.60	90	Almost 100	LED lamps	[66]
Mn/Fe-MOFs	Solvothermal	RhB	3 × 10^−5^ M	0.10	120	91.78	40 W LED lamps	[67]
TiO_2_@NH_2_-MIL-101(Fe)	Self-assembly	MB	50	0.20	30	96.00	300 W xenon lamp	[68]
MIL-100(Fe)/polymer	Photopolymerization	Acid Black	15 ppm	/	30	95.20	UV–Visible Light	[69]
M/Fe-MOF (M = Co, Cu, Mg)	Solvothermal	RhB	3 × 10^−5^ M	0.25	120	92.00	40 W LED	[70]
α-Fe_2_O_3_@C@SiO_2_/TiO_2_	Solvothermal	RY145 dye	50–250	0.30	90	100	8 W 12in T5 TUV	[71]
Fe-MOFs	Oil bath	MB	20	0.25	180	76.16	500 W mercury lamp	[72]
Fe_3_O_4_@GO@MIL-100(Fe)	Hydrothermal	2,4-DCP	50	/	60	Almost 100	500 W xenon lamp	[73]
Fe_3_O_4_@MIL-100(Fe)	In-suit growth	Levofloxacin	200	0.33	180	93.40	PLS-SXE300/300UV	[78]
NH_2_-MIL-101(Fe)	Post-synthetic modification	TBBPA	1.84 mM	0.50	120	Almost 100	/	[79]
PDINH/MIL-88A(Fe)	Facile ball-milling	CQ	10	0.40	30	94.60	PCX50C	[80]
CNT@MIL-101(Fe)	Hydrothermal	Ciprofloxacin	3.02 μM	0.50	60	Almost 100	White light LEDs	[81]
MIL-101(Fe)/γ-Fe_2_O_3_	Hydrothermal	OTC	25	/	60	91.20	Visible light	[82]
Fe_3_O_4_@MIL-53(Fe)	Calcination	IBP	10	0.40	60	99	500 W xenon lamp	[84]
NH_2_-MIL-53(Fe)	Solvothermal	CO_2_	50	0.002	5 h	CO/87.6 μmol·g^−1^ (6 h)	300 W xenon lamp	[127]
NH2-MIL-101(Fe)	Hydrothermal	CO_2_	50	0.10	8 h	CO/25 μmol·g^−1^·h^−1^ CH_4_/11.67 μmol·g^−1^·h^−1^	300 W xenon lamp	[128]
PCN-250-Fe_2_M (M = Mn, Zn, Ni, Co)	Solvothermal	CO_2_	/	/	4 h	CO/21.51 mmol·g^−1^ (4 h)	300 W xenon lamp	[129]
Fe-soc-MOFs	Solvothermal	CO_2_	50	0.05	5 h	CO/1804 μmol·g^−1^·h^−1^	Visible light	[130]
MIL-101(Fe)	Solvothermal	N_2_	80	0.05	60	Nitrogen fixation activity 50.35 μmol·L^−1^·h^−1^	300 W xenon lamp	[132]
MIL-101(Fe)	Microwave-solvothermal	NO_x_	0.1 ppm	0.10	60	77	150 W xenon lamp	[135]
Fe/Zr-MOFs	Solvothermal	N_2_	40	0.002	5 h	Nitrogen fixation activity 49.8 μmol·L^−1^·h^−1^	300 W xenon lamp	[136]
MO@Fe/Ce-MOFs	Calcination	N_2_	500	0.0025	2 h	Nitrogen fixation activity 299 μmol·L^−1^·h^−1^	500 W xenon lamp	[137]
Fe-MOFs	Solvothermal	VOCs	1000	0.20	2 h	CO_2_/460 ppm	INNOVA 1412i	[138]
MIL-100(Fe)/α-Fe_2_O_3_	Hydrothermal	VOCs	120	0.095	200	100	250 W xenon lamp	[139]

## Data Availability

Not applicable.
